# Novel instrumented frame for standing exercising of users with complete spinal cord injuries

**DOI:** 10.1038/s41598-019-49237-3

**Published:** 2019-09-10

**Authors:** Ioannis D. Zoulias, Monica Armengol, Adrian Poulton, Brian Andrews, Robin Gibbons, William S. Harwin, William Holderbaum

**Affiliations:** 1 School of Biological Sciences, University of Reading, Reading, UK; 2 School of Computing and Communications, Open University, Milton Keynes, UK; 3 Nuffield Department of Surgical Sciences, Oxford, UK; 4 School of Engineering, University of Warwick, Warwick, UK; 5 Aspire CREATe, University College London, London, UK; 6 School of Engineering, Metropolitan University of Manchester, Manchester, UK

**Keywords:** Bone, Biomedical engineering

## Abstract

This paper describes a Functional Electrical Stimulation (FES) standing system for rehabilitation of bone mineral density (BMD) in people with Spinal Cord Injury (SCI). BMD recovery offers an increased quality of life for people with SCI by reducing their risk of fractures. The standing system developed comprises an instrumented frame equipped with force plates and load cells, a motion capture system, and a purpose built 16-channel FES unit. This system can simultaneously record and process a wide range of biomechanical data to produce muscle stimulation which enables users with SCI to safely stand and exercise. An exergame provides visual feedback to the user to assist with upper-body posture control during exercising. To validate the system an alternate weight-shift exercise was used; 3 participants with complete SCI exercised in the system for 1 hour twice-weekly for 6 months. We observed ground reaction forces over 70% of the full body-weight distributed to the supporting leg at each exercising cycle. Exercise performance improved for each participant by an increase of 13.88 percentage points of body-weight in the loading of the supporting leg during the six-month period. Importantly, the observed ground reaction forces are of higher magnitude than other studies which reported positive effects on BMD. This novel instrumentation aims to investigate weight bearing standing therapies aimed at determining the biomechanics of lower limb joint force actions and postural kinematics.

## Introduction

The use of Functional Electrical Stimulation (FES) for rehabilitation and restoration of lost function for people with Spinal Cord Injuries (SCI) has been an active topic of research for over 50 years^1–9^. Originally research in FES restoration of function showed that it is possible to use FES for assisted standing aid in people with complete paraplegia^5,10^. Research has also demonstrated therapeutic benefits for FES (increased muscle mass, reduced risk of pressure sores, increased cardiovascular function). Among these benefits of FES in people with SCI, bone health has emerged as a topic of interest^11,12^.

People with complete SCI have decreased bone mineral density (BMD) in the lower body resulting from a total loss of sub-lesional muscle activity against gravity. The quality of health and life expectancy is reduced due to the risk of fractures aggravated by osteoporosis. In people without SCI loss of BMD is linked to a number of physiological factors including bone force loading by muscle contraction^13^; healthy bone is maintained through appropriate exercising and everyday use of the limbs with sufficient bone loading forces. Researchers have shown that these processes also apply to people with SCI using FES, whereby sufficient forces can be achieved to induce osteogenesis. Shields and Dudley-Javoroski showed this effect using repeated contractions of the plantarflexors with the leg isometrically constrained in a frame whilst sitting^14^. Malagodi *et al*. attempted to generate bone loading forces with more physiologically unconstrained exercises, using a standing frame and FES^12^. Such exercises may lead to more natural distribution of BMD. Using evidence from experimental results and biomechanical data^15^ Lambach *et al*. produce an empirical measure that connects changes in BMD in people with SCI with the magnitude and frequency of bone loading forces^16^.

In this paper, we describe an FES standing system which recreates and enhances the previous work on physiologically relevant bone loading against gravity while standing. The system enables the recording and analysis of postural and biomechanical information synchronously with real-time FES of lower body musculature. At the same time, visual feedback allows the user to react to the stimulated lower muscle, and interact through control of the upper limbs. The system’s data acquisition, programmable FES and biofeedback components are fully synchronised to enable experimental FES controls and biomechanical models to be safely and precisely compared.

This novel system offers improvement on the previously reported systems. Specifically, it introduces new techniques for full-body biomechanical, postural, and force monitoring, improving from the system described by Malagodi *et al*.^12^ allowing to record synchronised postural and ground reaction data from active FES standing. The side to side standing exercise aims to increase the observed ground reaction forces from the sitting exercising FES system as well as standing system where body weight is equally supported by each leg, by allowing much of the body weight to be supported on a single leg.

The main objective of the FES standing system is to enable repetitive bone loading exercises to be safely and effectively performed. The system aims to collect a variety of postural and biomechanical data so that exercises could be compared and assessed for their influence of BMD and improvement of motion range. Users of this system must comfortably stand and exercise for long periods with most of their body weight being supported by their lower limbs.

To validate the system’s performance, this paper presents biomechanical results from a case study on body weight support during side to side exercises conducted with 3 SCI volunteers over a 6-month period.

## Materials and Methods

The FES standing system comprises custom built hardware and software, a frame, and off-the-shelf sensors. The aim of this system is (i) to allow users with SCI to safely build lower limb muscle conditioning sufficient to perform standing exercises over hour-long sessions, and (ii) to simultaneously record biomechanically relevant information of body posture and the forces applied by the legs and arms of the user.

The design approach was iterative and incremental, with several stages of the standing system tested by expert users (healthy users and users with SCI) and in pilot experiments^17^ before achieving the finalised system detailed here.

### Instrumented frame

#### Design and construction

**Figure 1:**
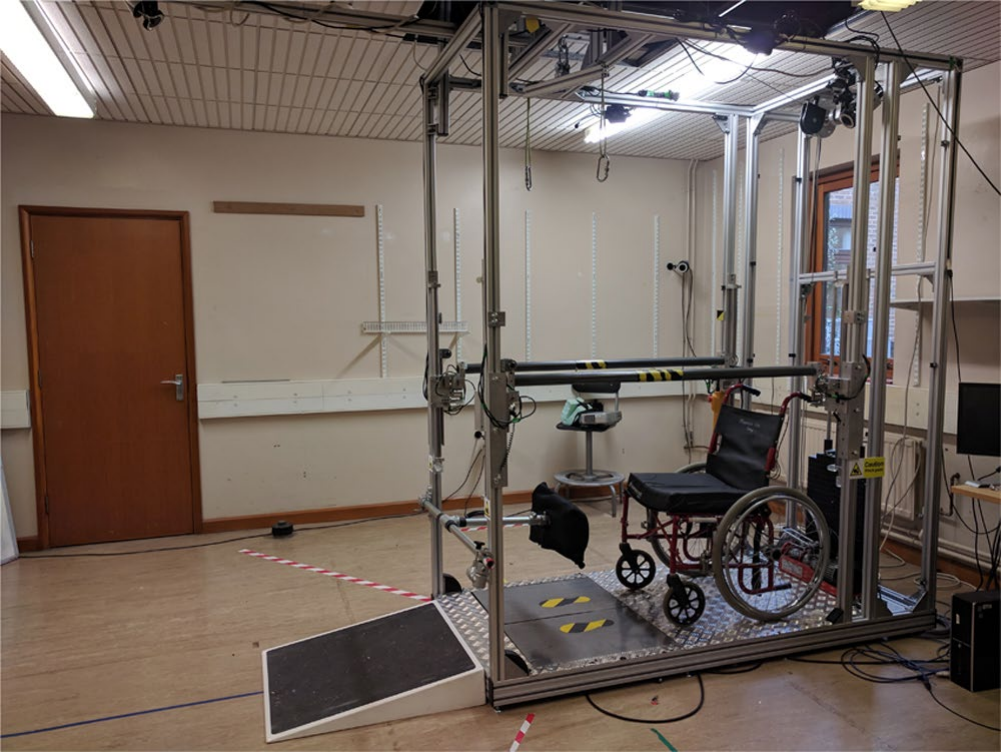
Standing frame. A Rexroth construction with two arm supports, a wheelchair entrance ramp, and the wheelchair used for sitting. An over-head winch powered mechanism holds a safety harness, aids with transfer to and from the wheelchair and a standing position. There are two force plates on the floor of the frame, each with an indicator for foot placement.

The frame was constructed using aluminium extrusions (Rexroth, Bosch Rexroth GmbH), metal tubes with plastic outer-casing for the arm support rails, and a tread-plated steel sheet for the flooring (see Fig. 1). The frame is sufficiently wide for a wheelchair carrying the user to enter the frame (internal measurements: (w) 84 cm, (h) 225 cm, (l) 172 cm). The wheelchair remains inside the frame for the duration that the user is standing. A ramp at the front of the frame allows the wheelchair to be lifted to the raised height of the frame. The arm supports are height adjustable (80–110 cm) to accommodate users of different heights.

#### Safety features

To ensure user safety during the standing exercises, a full body harness attached to an over-head winch mechanism supported the full weight of the participant during sit-to-stand and stand-to-sit transfers, to and from the wheelchair. The frame was fitted with a height adjustable knee pad with a protective foam and gel covering which provides passive standing support. Furthermore, a soft textile, back-support sling was attached to the frame and could be quickly engaged for additional weight support of the user, if needed, and/or for weight support during rest.

#### Sensors

The frame is equipped with sensors measuring ground reaction and hand bar forces, and recording body positions (load cells, force plates, and motion capture cameras). These sensors were used to assess the performance and biomechanics during the standing exercises performed by the user and provided feedback information which was displayed to the user in real time.

Load cells: Each of the two arm-support rails in the frame was equipped with 6 load cells (3A, Interface Force Measurements Ltd) which records the forces applied via the user’s hands in 3 Degrees-of-Freedom (DoF). For each force DoF, the average output is reported from two co-planar load cells situated at the opposite ends of the arm support; this achieved a balanced and stable force load on the arm support rail and eliminates torque at the attachment points of the rail to the bar. The load cells were used as the only attachment points of the arm support on the frame, thus recording all arm forces applied by the user’s arms. The analogue output of each pair of load cells was converted to a digital signal using a 4 input A/D bridge (PhidgetBridge, Phidgets Inc) which interfaced to the recording PC through USB. Each DoF was calibrated by applying weights in the range of 0–58.5 kg in 4.5 kg increments.

Force plates: Ground reaction forces were measured through two 6 DoF (3 DoF force, 3 DoF moments) analogue force plates (4060-10, Bertec Inc). Each plate was placed to record the forces and moments applied by one of the user’s legs. Two anti-slip foot shaped stickers were used to position each foot on the force plates and to ensure repeatable positioning. The force plates fed their output to a dedicated analogue amplifier for each plate (AM6501R, Bertec Inc). The amplified analogue output of the plates was in turn digitised and recorded by the recording PC using a USB A/D device (LabJack U6, LabJack corporation). The force plate calibration matrices provided by the supplier were validated by applying known weights (as per load cells, above) on each force plate.

Motion capture system: An 8-camera motion capture system (Miqus M3, Qualisys AB) records body posture and motion through 50 passive reflective markers (see Supplementary material Fig. 1). Three-dimensional positional data from each marker was derived from the camera images at a frequency of 100 Hz using an analysis software (QTM, Qualisys AB) which labelled each reflective marker based on a pre-determined model. The predetermined model was derived by sample data using the same marker positions in multiple users to provide a high accuracy on-line labelling for real-time use. Camera calibration was performed before every use, via a reference marker set provided by the supplier.

### Software

**Figure 2:**
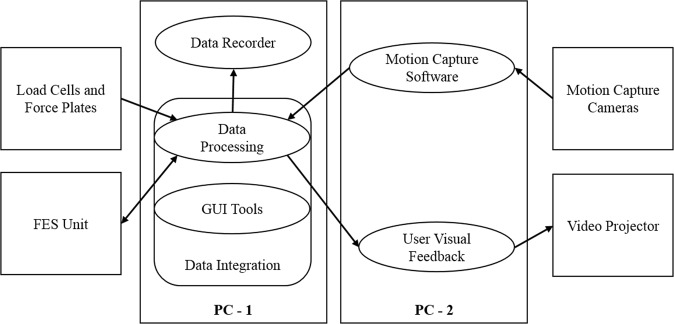
Data flow diagram between hardware and routines. The data processing routine receives data streams from the load cells, force plates, motion capture system and the FES device. Synchronised and processed data is then passed: (**a**) to the visual feedback to the exergame, (**b**) back to the FES device to control the stimulation output, and (**c**) to the dedicated data recorder for storage of the whole data stream. User interface options (GUI Tools) allow visualisation of the system status (e.g. if a sensor stops responding), modification of muscle stimulation parameters, and labelling of timepoints if necessary (e.g. timepoint when rest starts).

The standing system required various programs for operation: off-the-shelf software for motion recording (QTM); software we created for interfacing with force plates, load cells and the FES unit; and the user feedback program. Specialised software was developed to interface between all these and support real-time operation and synchronous co-recording of all the sensor data streams. To balance the load from processing, necessary third-party programs, and the visual feedback software, two PCs were used. The main PC was tasked with: (i) data integration, processing and recording, (ii) the control of output to the FES device and (iii) transmitting post-processed sensor output for use in the user feedback program. The secondary PC was used to display the visual feedback based on the commands from the main PC, and to run the motion capture software (see Fig. 2).

#### Real-time sensory integration and processing

A dedicated program for integrating, processing, and recording the data in real-time was developed in C#. The program used a timing library (MicroLibrary.cs^18^) to allow precisely timed events at a rate of 100 Hz. This program was composed of several subroutines which handled data saving, visualisation of system parameters and real time monitoring of sensor streams. Visualisation of system parameters and sensor monitoring was used for assessing standing during experiments and labelling events. Communication and transfer of data streams between the PCs, subroutines, and the dedicated data saving program (LabRecorder.exe) was achieved through the Lab Streaming Layer (LSL) library^19^. Using the LSL library allowed an abstracted design of information flow, with each of the programs and subroutines specifying the required streams that they needed to be subscribed to; this simplified the need for specifying IP addresses and creating bespoke communication protocols, whilst also providing accurate time stamping and synchronisation between data streams.

#### User feedback

**Figure 3:**
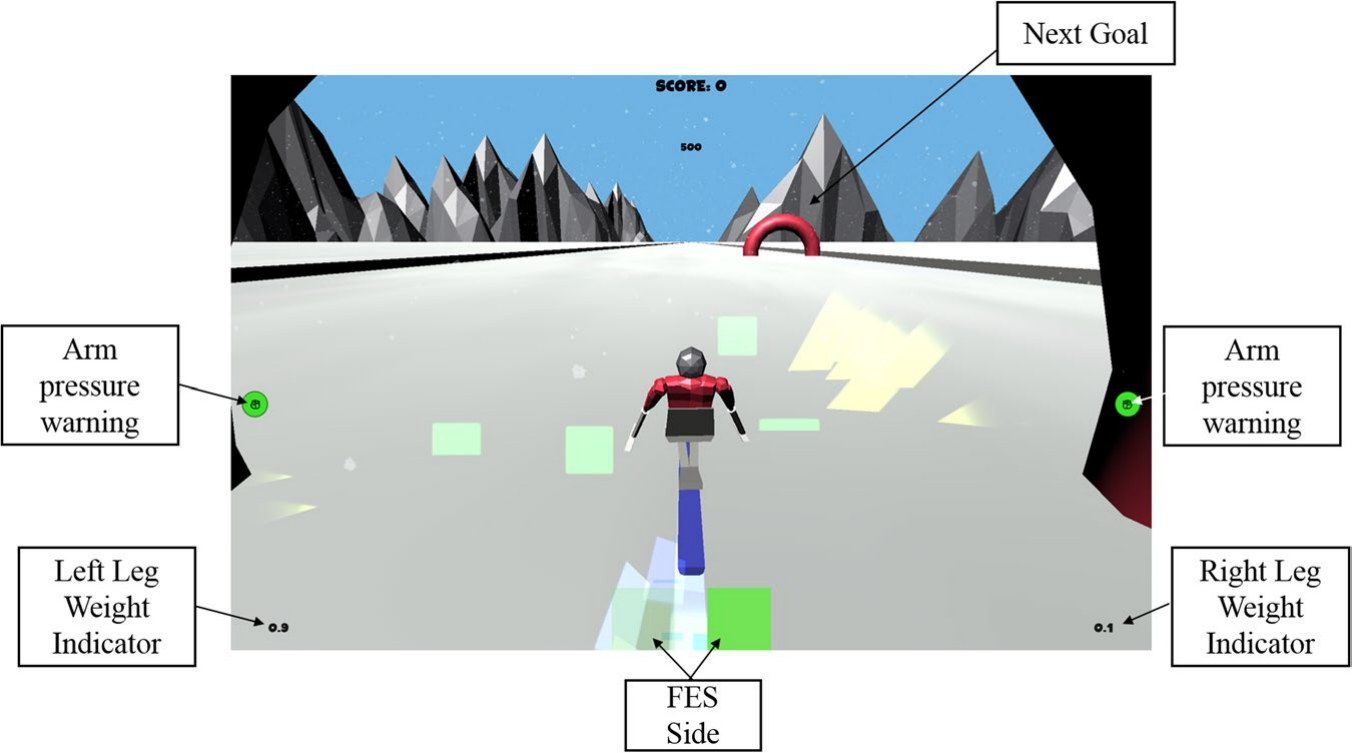
Exergame view during exercising. The virtual skier crosses a hoop as the next goal appear on the other side of the slope. Weight applied by each leg is shown at the corner in kilograms. An indicator at the bottom middle shows which side is currently being stimulated by the FES device. Annotated screenshot of the exergame^20^.

An exercising game (“exergame”), was developed in collaboration with Lincoln University^20^. The exergame, designed in Unity 3D (Unity Technologies), provided visual feedback of body posture and applied forces to the user during standing exercises (see Fig. 3). The exergame is a skiing game simulator, where the horizontal position of a virtual skier on the screen is controlled by the player’s bodyweight distribution in the standing frame. The goal of the user was to successfully pass through hoops which were presented at either side of the screen.

The exergame was displayed during the exercising via a projector on a screen 2 m directly opposite the user. The exergame presents the next postural goal as hoops on the left or right side of a slope, whilst the skier is constantly moving down the slope. To successful cross under the hoop the player must maintain their body weight on the correct side until reaching the hoop. Crossing the hoop in the middle awards the player points and a visual animation for successful crossing.

Other than the position of the skier, additional feedback information available to the user include: (i) body weight supported by each leg, (ii) indication of which body side FES is applied to, and (iii) excessive arm force indication when the player is relying on their arms for support.

The exergame was used to close the control loop between the muscle stimulation and the user’s posture; it encouraged the users to shift their upper body weight over the stimulated lower limb muscles, aiming to increase the effectiveness of the standing exercises through increased bone loading.

The inclusion of the exergame in the system aimed to satisfy two main goals: (i) allow the user to receive intuitive feedback during exercising, (ii) reduce the dullness of a very simple exercising task and increase user engagement^21^.

### Functional electrical stimulation device

We designed and constructed a purpose-built FES device for use in the standing system. The device was designed to supply high-amplitude, multi-muscle stimulation with controllable pulse width in real time, which was not available from other off-the-shelf devices at the time. The device was equipped with 16 output channels to allow for a wide range of lower-limb muscles to be stimulated at once. The device could be controlled through computer input and by a human operator with the control method changing seamlessly – this allowed the researchers to take over stimulation control during transfer to/from the wheelchair and rest periods. The device was powered by 8 AA batteries internally or by using a medically approved 2-stage mains-power separation 12 V power supply.

#### Output parameters

The FES device produces a 30 Hz output signal with pulse width being adjustable from 0–500 μs. The pulse amplitude is 138 mA for a token load of 1 kΩ parallel to 100 nF. These parameters were chosen to allow active standing for participants, allowing the lower muscles to contract sufficiently for the participants to sustain a standing posture for the duration of the experimentation^5,22^. The device frequency and amplitude is fixed during experiments, but can be adjusted through firmware updates. The pulse width for each channel is controllable through either the potentiometer knobs on the device or via digital communication to the COM signal from a PC.

#### FES pattern

A cyclical, posture shifting, FES pattern generator was implemented in order to provide efficient standing for long periods^2,5^. The aim of this routine was to produce prolonged standing for at least one hour, where each transition produced strong forces on the lower limbs, whilst using minimal hand forces for balancing and for upper body shifting.

**Figure 4:**
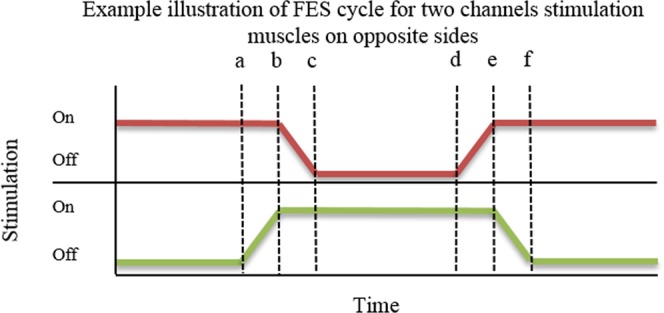
Illustration of FES pattern over time for a pair of muscles on opposite stimulation sides. (**a**) The green channel begins a switch ON transition whilst the red channel is still fully stimulating. (**b**) The green channel reaches full stimulation and the red channel starts transitioning to OFF. (**c**) The red channel is fully OFF. (**d**) The red channel begins transition to ON, mirroring step (**a**). (**e**) The red channel is fully ON, green begins transition to OFF. (**f**) The green channel is fully OFF. The ON level (i.e. the pulse width of the stimulation) is adjusted manually by the human operator of the device. The OFF level can be set as a percentage of the ON level so to provide support on the resting leg if necessary. In the experiment described in this paper, ON time was set to 5s, OFF time was set to 3s, and transition time was set to 1s.

A signal generator was programmed on the main PC for controlling the FES posture shifting routine with the following variables: ON time, OFF time, transition time, and OFF-stimulation percentage. The ON time corresponds to the length of time the stimulated side will remain stimulated, OFF time is the rest period for the non-stimulated side, transition time is the time it takes for a side to switch from stimulation to rest and *vice versa*. Finally, the OFF-stimulation percentage corresponds to the level of stimulation received by the muscles during rest, if any – a 50% level would correspond to half of the stimulation level that is delivered during the ON time being delivered during rest, whereas the 0% level indicates no stimulation at all during rest. An illustration of the delivered signal for a pair of muscles is shown in Fig. 4.

#### GUI for FES pattern

A graphical user interface was developed in C# for use with the FES device. The software allows the adjustments of the FES pattern parameters discussed above, and can assign each channel to one of the two stimulation groups. A visual representation of the current FES cycle location is shown to allow visual inspection of muscle contraction and confirmation of the electrode setup. This software was integrated in the GUI tools (see Fig. 2).

## Experimental Design

To validate the standing system, we present data from an experiment conducted over a six-month period which included a pre-experimental training period of approximately three months (in total over nine months). This experiment aimed to assess a simple side to side weight-shift exercise providing bone loading forces against gravity to the lower limbs of participants with complete SCI. The goal was to build sufficient lower limb conditioning to enable 60 min weight-shifting sessions.

### Participants

The inclusion criteria for the study were a complete SCI between T1-T10 with a positive neurological response to electrical stimulation of the lower limb muscles. Ability to passively stand on a standing frame was also required. Prior to beginning the standing exercises, participants trained with an FES device at home completing alternate knee extension exercises for 60 min, 3–5 times a week. Standing commenced when participants could perform full knee extensions with 1 kg weight resistance at the ankle for 60 min. Of the 10 participants originally recruited, only three participants completed the 3-month training period (see Table 1).

**Table 1:** Participant Details.

Sex	Participant 1	Participant 3	Participant 5
	M	M	M
Age	36	34	33
Weight (kg)	83.3	72.7	57.2
Injury Level	T4	T4	T2
AIS	A	A	A
Years post Injury	1	15	4
Number of Sessions	36	36	41

This study was approved by the University of Reading ethics committee and the NHS research ethics committee. All methods described in this paper were conducted in accordance with the approved guidelines. Participants were provided with information about the purpose and procedures of this study, and were informed that the data and media recorded (presented in this paper) would be published in an online, open-access journal. Participants gave informed written consent to take part to the study and for the data presented in this paper to be published in an online, open access journal. Participants were compensated at £12/hr for their time.

### Experimental procedure

Each participant was scheduled twice weekly for a 2 hr visit comprising 60 min standing exercising and approximately 60 min for preparation and termination of the experiment.

At the beginning of each session, surface electrodes (5 cm diameter circular hydrogel electrodes, PALS, Axelgaard Manufacturing Co) were placed on the participant’s calf (gastrocnemius and soleus) quadriceps (rectus femoris and vastus lateralis), and gluteus maximus muscles. The exact electrode position for each participant was determined after identifying the each muscle’s motor point, using a motor point pen (Compex motor point pen). A safety harness was then worn by the participant who would enter the standing frame seated on the wheelchair. The retractable kneepad would then be secured locked in place.

In sessions where motion capture data were recorded (approx. once a month) the passive markers were attached to the participant using double sided tape (Supplementary material Fig. 1). Motion capture recording session were restricted due to timing limitations for the experiment; adding motion capture would increase the experimental time by approx. 40 min due to extra preparations necessary. This included positioning and securing the markers, repositioning then after the user was standing on the frame (markers moved during the transfer), and removing the markers at the end of the experiment.

When ready to begin, the participant adopted a standing posture through: a) upper body support on the hand rails, b) incremental manual application of FES on the quadriceps, and c) weight lifting assistance from the safety harness by raising the over-head winch. When the participant could hold a stable standing posture, the harness was lowered such that it did not provide weight bearing support. At that stage, FES output would change from continuous to the posture shifting pattern as described earlier. For the cyclical stimulation, muscles from each side (i.e. left and right lower limbs) were grouped together to support the side to side exercise. The stimulation ON period was set to 5s, with transition time being 1s and OFF period equal to 3s. Thus, a single exercise cycle is defined as: (i) starting at the centre, where body weight equally is supported by both legs (upper body is in a straight posture), (ii) moving to one side (left or right) and holding for approx. 5s, and (iii) returning back to the centre to start a cycle for the opposite side.

Following the above stimulation pattern, participants exercised for 60 min, using the exergame visual feedback to coordinate upper body movements with the FES pattern.

### Data analysis

Presented in this paper is a summary of data from a six-month longitudinal study that investigates the effect of load bearing on the health of bones.

Data were imported, analysed, and plotted in Matlab v. 2014b (MathWorks inc). To find the forces during the left/right side to side postures, a function for finding local maxima was used on the raw force data recorded in the Z dimension (against gravity) from the force plates. Local maxima were required to have an amplitude of at least 50% of body weight to qualify as a peak. A separation distance of 5s between peaks was used to exclude counting multiple local maxima within a single cycle. In combination, these two measures excluded any artificial peaks (during the early and late sit-to-stand and stand-to-sit periods) counting only the true peaks during exercising. Positive peaks from the forces of each plate corresponded to the maximum force applied during the posture shifting to the respective side. Peaks reported by the algorithm were visually inspected to eliminate counting abnormal peaks and to ensure the correct application of the inclusion criteria (e.g. at the edges of the data, counting the same peak twice).

Based on the timing of each peak, the co-recorded forces and posture data from all other sensors were collected. This allowed observation of the distribution of the forces during the peak of the posture shift and counting the number of side to side exercise cycles performed during a session. Furthermore, it allowed tracking of the peak forces produced within and between sessions. Participants’ weight was periodically measured in the standing frame by subtracting the wheelchair’s weight from the recorded combined weight of the participant and the wheelchair. Using this recorded weight, forces were converted to body weight percentage per participant by dividing by each participant’s recorded weight. A value of g = 9.81 m/s^2^, where g is the standard acceleration due to gravity, was used to convert forces from newtons to kg.

For the posture data, the clavicle marker (see “Clav”, Supplementary material Fig. 1) was used to provide the range of motion as an angle: this was derived by the arctangent of the measured marker coordinates, with a result of 0° corresponding to the start of the exercise cycle, as defined above. Motion range was derived by addition of the absolute angle of successive cycles (i.e. the angle reached by left lean cycle added to the angle reached by the following right lean cycle).

### Statistical analysis

Statistical analysis was also conducted in Matlab. Data were summarised as a table of means of the derived values (i.e. peak body weight percentage or side to side motion range) from each session, grouped by session number (i.e. early or late). The design was set as within-participant with repeated measures; repeated measures analysis of variance (r-ANOVA) and Tukey’s test was used for omnibus and post-hoc comparisons, respectively^23^. The results presented below report the F-statistic given the degrees of freedom of the factor (i.e. time measured by session number) and the residuals. P values reported are precise to 4 decimal points.

## Results

### Standing time and exercise repetitions

After leg conditioning at home was complete, participants were asked to stand on the instrumented standing frame using FES. Participants managed to complete a full 60 min standing posture with weight shifting by the second session. Each participant completed at least 35 sessions. Within the first 5–8 sessions participants could reach the maximum number of exercise cycles that can be achieved in a 60 min training session (i.e. approx. 650–700 repetitions), with the weight to the supporting leg being at least 60% of body weight (mean weight on the supporting leg across participants after the 5^th^ session was 75.32% of body weight, standard deviation (SD): 7.32%, standard error of the sample mean (SE): 0.73%, see also Supplementary material Fig. 3).

### Body weight distribution within-session

**Figure 5:**
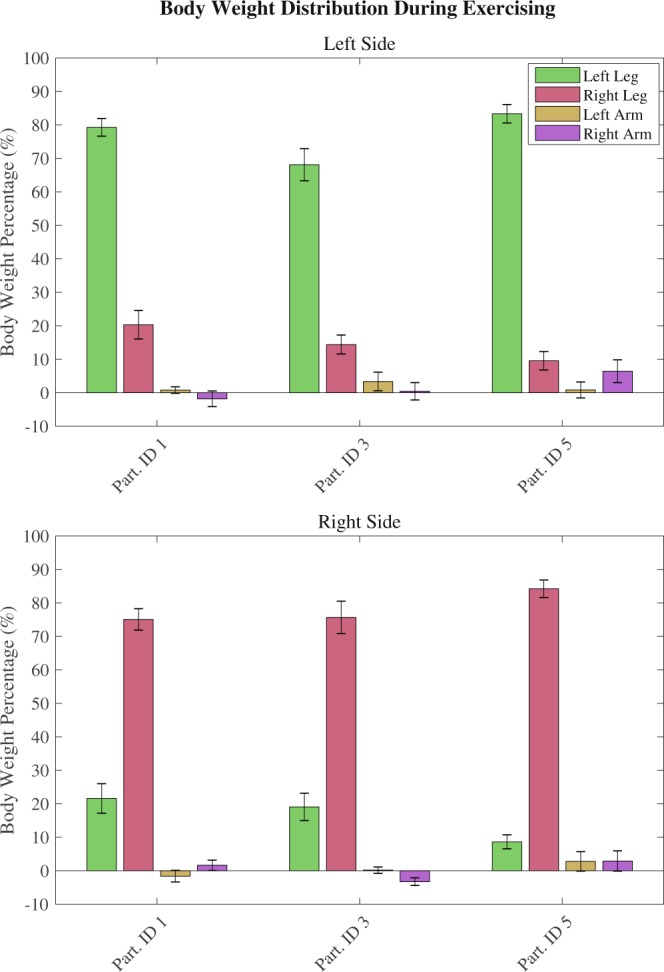
Distribution of peak forces across different limbs for each participant. Upper: forces distribution on all limbs during left-side leaning cycle. Lower: right-side leaning cycle forces. Most of the body weight is distributed to the supporting leg for each side, with the arms providing little support. Negative values by the arm forces signify a pull action from the user (instead of pushing down on the arm support). This shows that participants employed different techniques to stabilise their upper body during exercising. Lines overlaid on the bars correspond to the standard deviations from the mean. Sample size for each bar is approx. 5000 exercise cycles.

Participants could exercise with forces being distributed mainly to the sided leg for each cycle (i.e. the leg which was stimulated). During the peak of the posture shift participants could apply most of their body weight on the supporting leg: a small percentage of body weight was supported on the OFF-side leg and less than 10% of body weight being supported by the upper body (mean arm support across participants after the 5^th^ session was 7.59% of body weight, SD: 5.05%, SE: 0.5%, see Fig. 5). In total, participants could support almost all their body weight with little aid provided by external supports (e.g. knee support).

It was observed that the force on the supporting leg remained stable during the ON period of the cycle (i.e. during maintaining the body weight on the stimulated leg). Similarly, upper body forces were stable during the same period, with changes occurring only during weight shift transition. This result is consistent with visual observations during the exercising session of participants maintaining a stable posture without exerting upper limb support forces, rather, only using them to maintain balance.

**Figure 6:**
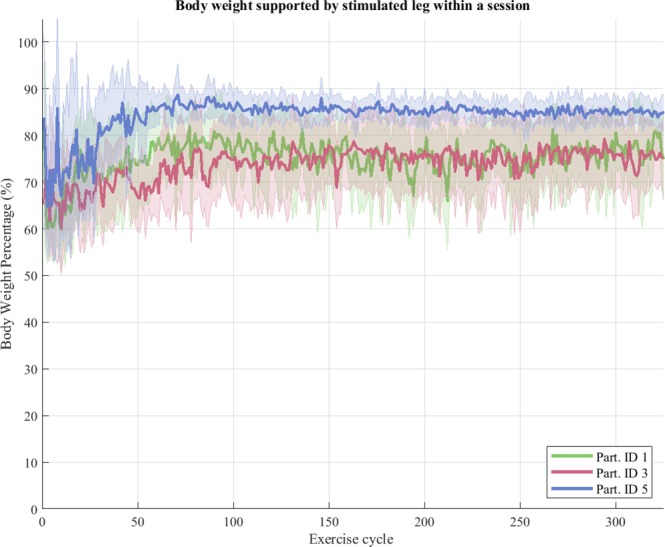
Within session peak leg support forces during successive cycle repetitions, for each participant. After a habituation period, leg support forces remain stable for the duration of the session. Shaded areas correspond to the standard deviation from the mean. Sample size for each time-point (exercise cycle) is 10 sessions.

Over the duration of the standing session, the peak force on each supporting leg was shown to be stable at or above approx. 70% of body weight with no discernible drop or decline due to fatigue (see Fig. 6).

### Exercise improvement over time

**Figure 7:**
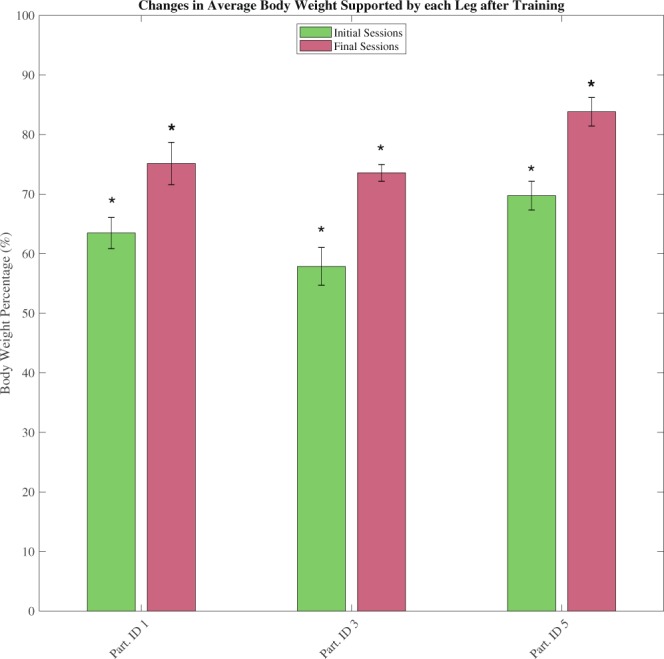
Average (mean) body weight distribution on the supporting leg between initial and final sessions (data from both left and right leaning cycles, forces supported by stimulated leg only). Mean (session mean) body weight distribution is significantly larger on the five final sessions than on the initial five (p = 0.0071, mean difference 13.88 percentage points). Lines overlaid on the bars correspond to the standard deviation from the mean. Sample size for each bar is taken from 5 sessions and approx. 3000 exercise cycles.

Exercise output measured by the body weight supported by the stimulated leg and the achieved range of motion was tracked over the duration of the experimental period. Of interest were any changes due to the long-term use of the standing system. Comparing body weight support data from the first and last five sessions shows an increase in the supported body weight (mean supporting weight on the early sessions across participants was 63.69% of body weight, SD: 5.63%, SE: 1.45%, on the late sessions 78.61% of body weight was supported on each leg, SD: 5.37%, SE: 1.12%, see Fig. 7). A repeated measures analysis of variance (r-ANOVA) showed significance in the body weight supported by each leg due to the session number F(9,18) = 22.293, p < 0.0001. A follow-up Tukey post-hoc test showed that the group of the final sessions had a significantly higher body weight supported on the stimulated leg than the initial sessions group p = 0.0071. The mean increase per participant was 13.88 percentage points of body weight supported on each participant’s later sessions than their first sessions.

**Figure 8:**
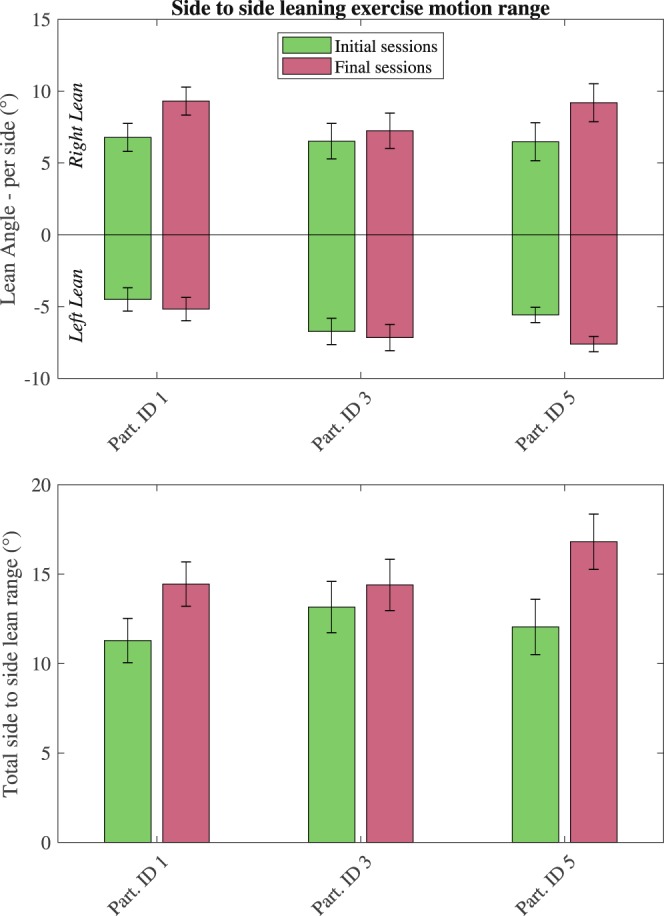
Range of motion from side to side exercises between early and late sessions as recorded by the motion capture system. Means are increased across all participants between early and late sessions, however, multiple comparison test showed no statistical significance (r-Anova:, p = 0.0316, Tukey post-hoc: p = 0.0966). Lines overlaid on the bars correspond to the standard deviation from the mean. Sample size for each bar is 2 sessions and approx. 600 exercise cycles.

The range of motion was also tracked between the early and later sessions. The mean range of motion, as tracked by the clavicle marker, was shown to increase between the early and later sessions (mean range of motion across participants on the early sessions was 12.13°, SD: 1.21°, SE: 0.49°, on the late sessions mean range of motion was 15.22°, SD: 1.26°, SE: 0.52°, see Fig. 8). A r-ANOVA showed significance in the total range of motion for the exercises due to the session number F(3,6) = 5.9259, p = 0.0316. However, the follow-up Tukey post-hoc test showed no significance, p = 0.0966, between the early and late sessions on the range of motion.

## Discussion

The results show that the standing system developed is successful in delivering repeatable side to side weight-shift standing exercise in participants with complete SCI. Peak performance, in terms of completing a session with continuous exercising, was achieved after a period of 5–8 sessions.

Of significance, it was shown that the forces produced at the beginning of a session were maintained throughout the activity. The data collected confirm that participants can produce side to side weight shifting exercises with the stimulating leg supporting the majority of body weight. The support forces on each stimulated leg were maintained throughout the stimulation (i.e. approx. 5 sec). In this regard, the system has achieved its initial goal of providing a safe experimental platform for studying standing exercising therapies. Moreover, the lack of a decline in the leg support forces shows that longer standing sessions with this platform would be possible without any further changes to the protocol required.

In this paper we observed larger ground reaction forces compared to other systems which have reported BMD increase through load bearing exercising. In a study by Lambach *et al*., the effect of bone loading (during FES-assisting rowing) on BMD was reported^16^. Lambach *et al*., reported positive effects on BMD due to bone loading with observed average ground reactions forces between 17% to 26% BW. In this paper we report average ground reaction forces of 70% to 80% BW applied to a single leg during exercising. This substantial increase of applied forces reinforces the potential in using the standing system reported in this paper to study BMD improvements for users with SCI.

The functionally similar platform developed by Malagodi *et al*. did not report results from SCI participants using the platform^12^. In a study of ground reaction forces in patients with SCI during passive standing, K A Bernhardt *et al*. observed ground reaction forces of 70–90% BW supported by both legs^24^. Our results confirm these observations, with participants supporting approximately 90% BW on their legs. Crucially, the distribution of those forces during the sided-exercise is substantially on the leg currently being exercised (over 70% BW). This is in contrast to simply standing where the supported BW is equally split between each leg. To our knowledge, this paper presents the first results on ground reaction forces achieved when performing FES-assisted standing exercises by users with complete SCI.

Whereas the work by Shields *et al*., showed BMD effects by targeting a single muscle and creating a torque on the tibia^14^, our system stimulated a number of lower body muscles and used the participant’s body weight aiming to increase the bone loading forces. However, the forces recorded from the foot plates and presented in this paper must be translated to the equivalent bone loading forces, to assess the efficacy of the side to side exercise, as described by Shields *et al*.^14^. This is a limitation of this study, as higher ground reaction forces do not necessarily correspond to higher bone loading forces for the targeted bones. Current work in biomechanical modelling of the force and postural data will be used for this purpose^25^.

In this study we presented data from three participants, reporting a drop-out rate of 70%. This is not atypical, with similar studies reported having such limitations in the number of participants^26^. Using the standing frame requires a defined level of upper-body strength by participants; a larger pool of participants would better assess the suitability of the standing frame for the wider population of people with SCI.

Further to the ability to achieve standing exercises against gravity, the system showed an increase in the exercise output (body weight supported by stimulated leg and motion range) between early and late period experimental sessions (see Figs 7 and 8). Although the statistical significance of these improvements must be considered through the lens of the limited participant number and the importance of the exercise output increase must be assessed through other means (e.g. a bone loading model, pre and post intervention biophysical measurements) the side-to-side weight shifting exercise has validated the ability of the standing system to support and assess such exercises through a variety of sensory modalities.

An important point concerns the underlying factors that caused the observed improvements in the exercise output. Muscle conditioning, familiarisation with the apparatus and the exercise, and upper body balance improvement whilst standing, would allow participants to perform better after successive sessions. Moreover, the visual feedback provided motivation for the participants to improve their exercise output; participants could monitor the weight supported by the stimulated leg during each cycle and actively tried to improve. The exergame provided an interactive challenge by requiring the participants to increase the weight supported on the stimulated leg in order to achieve the goal (pass under the hoop). FES posture shifting is a technique that has long been used for standing individuals with SCI^5^. The weight shifting exercise presented in this paper is similar to the method of “posture switching” proposed by Kralj *et al*., but based on faster shifting times and shorter transitions of weight from the participant of less than 1s. The visual feedback and coordination from the participant are similar to the method presented by Sayenko *et al*., used for balance training of participants with incomplete SCI^27^. In our study, the control loop was closed by the human-in-the-loop; visual feedback steers the user to shift the upper body to the required side. The real-time sensor information processing nature of the platform lends itself to more sophisticated closed-loop control designs which have been suggested in the literature^28–31^. Introducing close-loop control designs which adjust the FES output based on posture, training goals, and neural feedback from the user could further increase the percentage of body weight supported by each leg at a time, and allow the users to attempt more extreme and/or dynamic postures (e.g. one leg standing, or squatting). Such improvements on the system could allow the targeting of different bones throughout the exercising routine and maximize the bone-loading forces for each targeted bone.

## Conclusions

In this paper we described a novel FES standing exercise system for SCI users which was validated as a platform for investigation of bone health. The standing frame improved work from previous systems, allowing synchronous data recording of postural and ground reaction data with real-time user feedback. Additionally, the system was validated through a case study of side to side exercises, allowing greater body weight percentage to be supported by each leg than previously reported.

The system allowed users with a range of SCI to perform 60 min standing exercise sessions with most of the forces distributed to the lower limbs and little external support. We showed that the participants’ exercise performance, as recorded by the biomechanical measurements by the standing frame, improved significantly in the duration of the study. These achievements met the original goals of creating a novel system for supporting the investigation of FES standing exercises safely and effectively for users with SCI.

This paper presents new results on the magnitude and distribution of forces and postural measurements during FES-assisted standing exercising for people with complete SCI. Importantly, we showed that by using the system, users generated ground reaction forces of substantially larger magnitude to other FES exercising systems which reported effects on BMD.

Findings from this work are increasingly important, as new techniques (such as stem cell implantation^32,33^, and brain-computer interface rehabilitation^34^) have shown promising results in people with SCI. Improving the lower musculoskeletal system is therefore essential to take advantage of such future possibilities to enable increased upright activities to be without risk of low trauma fractures.

## Supplementary information

Supplementary Information

FES Exercising - Overview

FES Exercising - Detail

## Data Availability

The data recorded during the current study are available from the corresponding author on reasonable request.
