# Supplementary material for: Novel instrumented frame for standing exercising of users with complete spinal cord injuries
Source: Sci Rep. 2019 Sep 10;9:13003. doi: 10.1038/s41598-019-49237-3 (PMC6736978; doi:10.1038/s41598-019-49237-3)
Supplement: Supplementary file 1 — Supplementary Information [file 41598_2019_49237_MOESM1_ESM.docx]

Novel instrumented frame for standing exercising of users with complete spinal cord injuries

I. D. Zoulias, M. Armengol, A. Poulton, B. Andrews, R. Gibbons, W. S. Harwin, W. Holderbaum


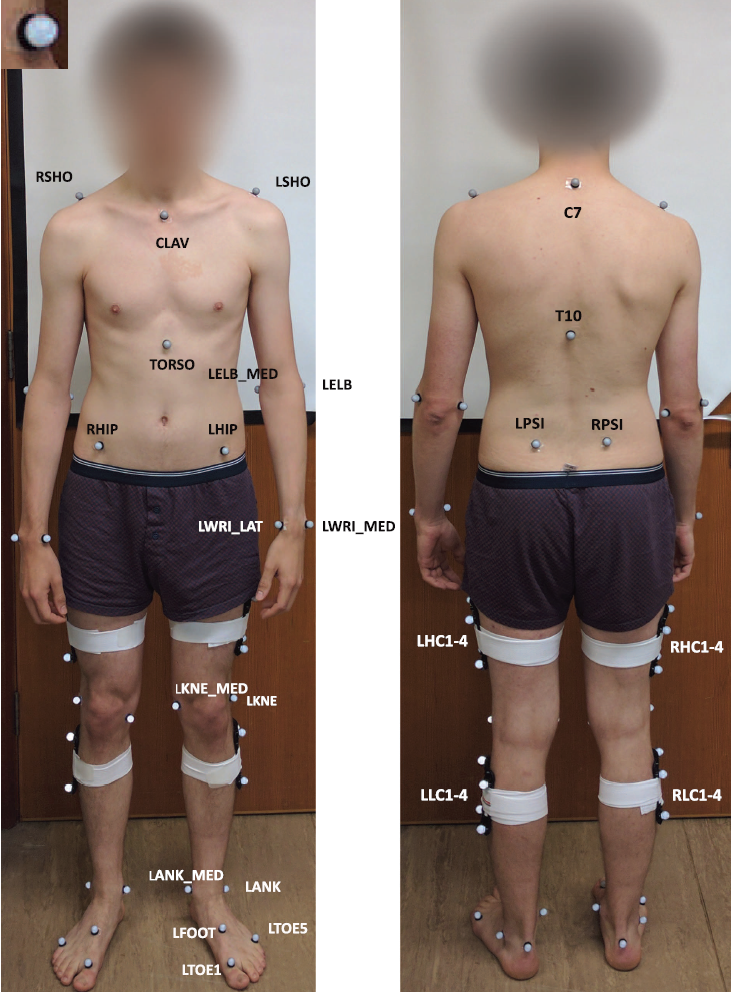


Supplementary Figure 1. Position of reflective passive markers on the body. Labels used for real-time and off-line analysis of body posture.


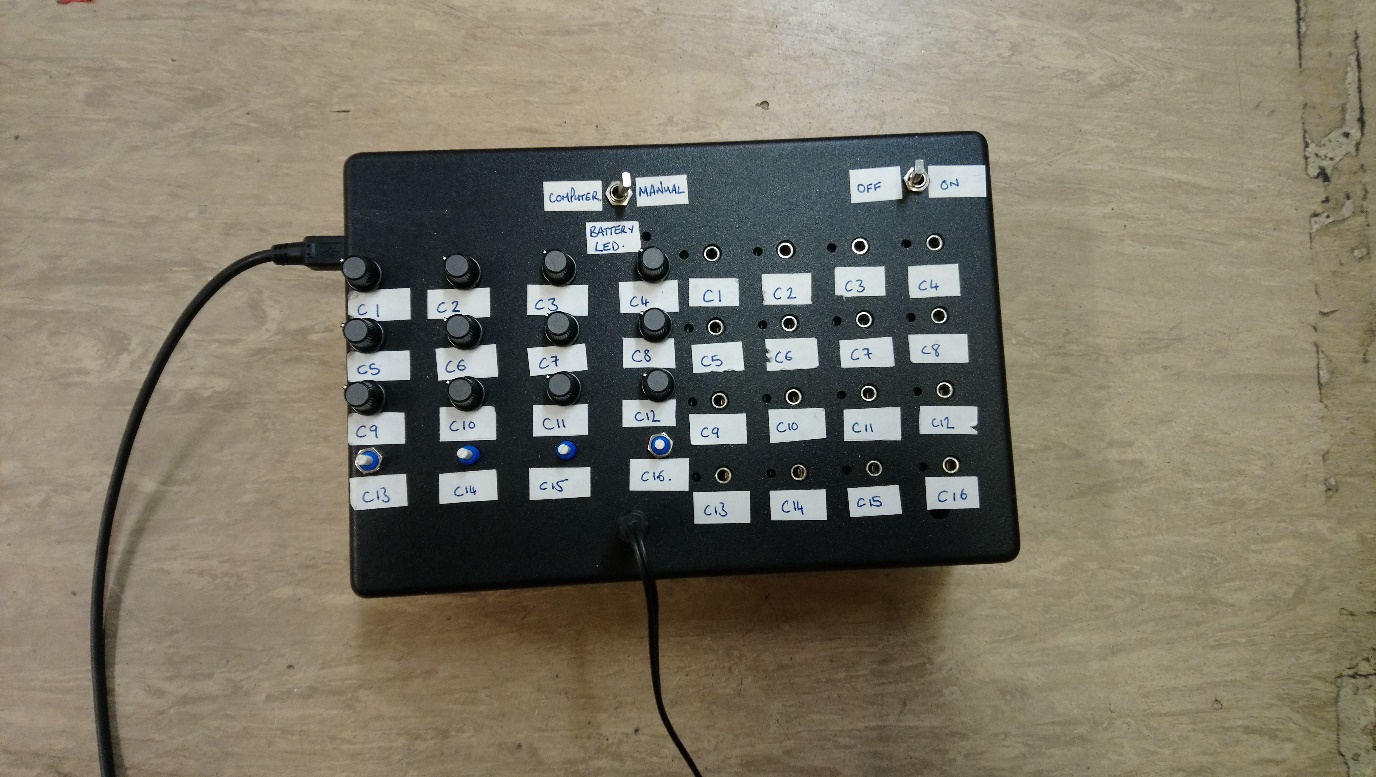


Supplementary Figure 2. FES device. The 16 output channels on the right can be controlled by the matching control knob on the left. Change between computer control or manual control can be made by the left switch at the top of the device.


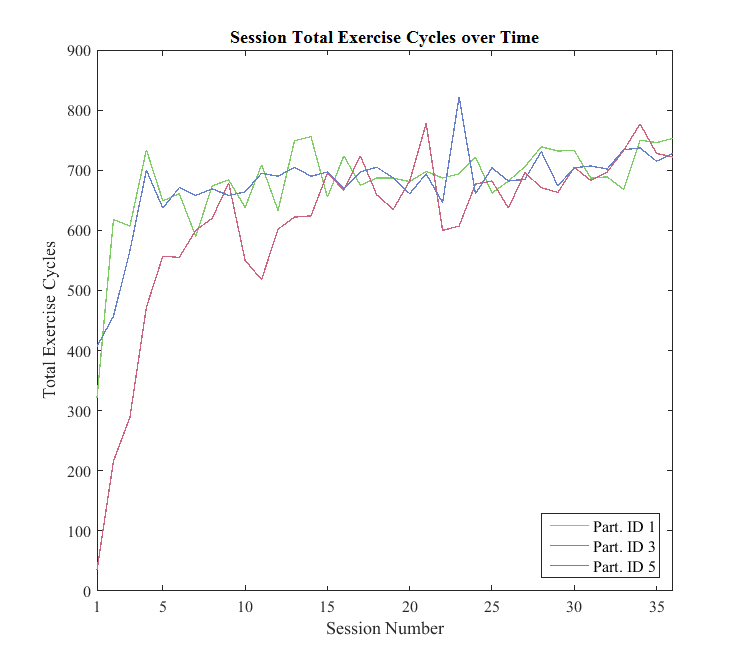


Supplementary Figure 3. Total exercise cycles performed within a session from each participant over progressive sessions. Within the first 5-8 sessions participants were able to achieve approx. 650-700 cycles and maintain that exercise output (continuous exercise for 60 min)
